# Gross Morphology and Histology of Head and Salivary Apparatus of the Predatory Bug, *Rhynocoris marginatus*

**DOI:** 10.1673/031.012.1901

**Published:** 2012-02-08

**Authors:** S. Muthu Kumar, K. Sahayaraj

**Affiliations:** Crop Protection Research Centre, Department of Advanced Zoology and Biotechnology, St. Xavier's College (Autonomous), Palayamkottai — 627 002, Tamil Nadu, India

**Keywords:** entomophagous, extra-oral digestion, reduviids, salivary gland, stylet

## Abstract

*Rhynocoris marginatus* Fabricius (Hemiptera: Reduviidae) is an important biological control agent against more than 25 insect pests in India. For a better understanding of the feeding adaptation of this bug, the gross morphology and histology of its head and salivary apparatus were studied using both a light microscope and scanning electron microscope. The head is more or less elongate, mobile, and immersed into the eyes. *R. marginatus* has a three-segmented curved rostrum; the middle segment is longer than the other two segments. The terminal rostral segment bears spines and trichobothria externally. Stylet bundles bear two pairs of maxillary and mandible stylets in the curved rostrum with serrations. The stylets help to penetrate into the tissue and directly pump the toxic venomous saliva deep into the prey. The principal gland is bi-lobed (anterior lobe and posterior lobe), whereas the accessory gland is uni-lobed, exhibiting distinct functional and histological differences. These glands receive tracheal and nerve supply. Mononucleated, binucleated, trinucleated and polynucleated cells are distributed both in anterior and posterior lobes of the principal gland. The cytoplasm has collecting vacuoles with secretions. Therefore, this predator is highly equipped with well-developed mouthparts that are attached to the salivary apparatus.

## Introduction

Reduviids (Hemiptera: Reduviidae) are predacious insects of agricultural importance as they act as biological control agent against many economically important pests. They are abundant in agroecosystems, semiarid zones, scrub jungles and tropical rainforest ecosystems (Ambrose 1999; Sahayaraj 2007). Their success in every ecosystem or trophic niche is due to their morphological and physiological adaptations in predation and extra-oral digestion. The reduviid predator, *Rhynocoris marginatus* Fabricius (Hemiptera: Reduviidae) is an entomophagous insect distributed in many agro-ecosystems, feeding on more than twenty economically important insect pests in India (Sahayaraj 2007). The potential of *R. marginatus* as a biocontrol agent under laboratory (Sahayaraj 2000; Sahayaraj and Balasubramanian 2009; Sahayaraj et al. 2003, 2004) and field conditions (Sahayaraj 1999; Sahayaraj and Martin 2003; Sahayaraj and Ravi 2007) has been previously reported.

The structure and function of the rostrum and salivary systems of hunter heteropterans have attracted increasing attention because of their prey-killing ability. However, the salivary system of predatory reduviids has not been given due consideration. The salivary system of reduviids conforms to the general heteropteran plan (Southwood 1955; Louis and Kumar 1973; Haridass and Ananthakrishnan 1981; Morrison 1989; Ambrose and Maran 1999; Sahayaraj et al. 2010). The morphology of salivary glands is diverse in different subfamilies, which could be utilized as a reliable taxonomical tool (Louis and Kumar 1973). The principal gland is uni-lobed, bi-lobed, or multi-lobed, whereas the accessory gland is unilobed and vesicular, exhibiting distinct functional and histological differences. The principal gland is divided into anterior lobes and posterior lobes, suggesting the differential functions of the lobes involving division of labor (Haridass and Ananthakrishnan 1981) with histological variations. The anterior lobes of principal glands secrete zootoxic enzymes used to paralyze the prey, whereas the posterior lobe secretes digestive enzymes. The accessory gland is typically vesicular (Southwood 1955; Edwards 1961), and differs histologically from the lobes of principal glands and secretes watery saliva (Haridass and Ananthakrishnan 1981; Morrison 1989) used in the lacerate flush mode of feeding in reduviids (Miles 1972).

A high number of reviews are available on the salivary gland structure of blood sucking Triatominae and other hemipteran predators. However, information on the functional morphology of salivary glands of entomosuccivorous reduviids is limited, with a few exeptions: Haridass and Ananthakrishnan (1981) on *Haematorrhophus nigroviolaceus*, *Lestomerus affinis,* and *Peirates affinis*; Sivaraj (1986) and Morrison (1989) on *Acanthaspis pedestris*; Santha (1986) on *Catamiarus brevipennis*; Udayakumar (1986) on *Ectomocoris tibialis*; Vellingirinathan (1986) on *Lophocephala guerini*; and Sahayaraj et al. (2010) on *C. brevipennis.* The study of the functional morphology of the salivary gland in reduviids is essential before the incorporation of salivary venom in toxicological and biochemical studies. Because detailed functional morphology of the salivary gland and other feeding apparatus of *R. marginatus* are not present in the literature, we aim to elucidate the gross morphology, functional morphology and histology of the salivary gland complex and supportive organs of *R. marginatus.*

## Materials and Methods

### Reduviid collection and rearing

Laboratory colonies of the *R. marginatus* were established from individuals that were collected from Tiruneveli district, Tamil Nadu, India. *Rhynocoris marginatus* were reared on larvae of the host, the rice moth, *Corcyra cephalonica* at 30 ± 2 °C, 70–80% RH, and with a photoperiod of 13: 11 L:D in round plastic containers measuring 7 cm height and 6 cm diameter.

### Sample preparation

*Rhynocoris marginatus* adults (n = 6–10) were anaesthetized by placement in a deep freezer for 5–10 minutes. The anaesthetized insects were sacrificed and used for this study. In another study, six to ten heads, including mouthparts, of *R. marginatus* were placed in 2.5% glutaraldehyde in 0.1 M Phosphate Buffer (pH 7.2) for 24 hours and then airdried. The heads were washed four times in buffer and three times in distilled water; each washing cycle lasted 15 minutes (Heng-Moss et al. 2003). Then, heads were dehydrated in 50, 70, 80, 90, 95, and 100% gradient ethanol for twenty minutes each. The whole head and stylet bundles (mandibular and maxillary stylets) were kept together by placing a minute pin at the base of the stylets. Specimens were coated with a 20 nm thickness of carbon with a sputtering device and viewed in secondary emission mode in a Hitachi S-2250N scanning electron microscope (Hitachi, www.hitachi.com) at 10 KV. Digital images were captured and stored in an IBM-PC (IBM, www.ibm.com) compatible computer.

### Gross morphology of mouthparts

At least ten specimens of *R. marginatus* were examined for gross morphology of mouthparts. The heads were dissected from the insects and mounted on honey wax blocks with fine pins. Both the labium and labium were removed, and the stylets were separated with a fine needle or a very small (0.01 mm) camel hairbrush moistened with distilled water. The approximate size of the base, middle, and terminal segment of rostrum and length of mandible and maxillae also were measured. Camera lucida diagrams of the entire head, maxilla, and mandible were performed using an ocular and stage micrometer. Microphotographs of the head and terminal of the rostrum were taken in scanning electron microscope as described above. Moreover, phase contrast and dark field photographs were taken using Olympus CX-41 Phase contrast microscope (Olympus, www.olympus.com). Ten to fifteen insects of the same age group were used for the analyses.

### Morphology of the salivary gland

The dissected glands were fixed for one hour in 0.1 M sodium phosphate buffer (pH 7.2) containing 40% (W/V) paraformaldehyde at room temperature. The glands were settled onto slides and studied with a trinocular Olympus microscope. The approximate length and diameter of the principal gland (anterior and posterior parts separately) and accessory gland were measured. Camera lucida micro drawing was performed.

### Histology of the salivary gland

The legs, wings, and tergal plates of the immobilized predators were carefully removed, and a circular lateral incision around the abdomen was made with a No. 11 Lister sterile surgical blade (Lister Technologies, www.listertechnologies.com) in insect Ringer's solution (0.0014 mol^-1^ NaCl; 0.0000413 mol^-1^ KCl; 0.25 moL^-1^ CaCl_2_; 0.25 mol^-1^ Na_2_CO_3_) (Lacey 1997). The main salivary duct was detached from the sclerotised mouthparts closer to the salivarium. The salivary gland complex was dissected out and placed in a clean watch glass in a drop of insect Ringer's solution. After a sufficient number of salivary gland complexes had been collected (> 10), the insect Ringer's solution was drained off and the glands were quickly washed in a few drops of distilled water. These were then drained away as thoroughly as possible and were fixed in alcoholic Bouin's solution. After 24 hours of fixation, the glands were dehydrated in a series of increasing alcohol dilutions (30, 50, 70, 90, and 100%), for 15–30 minutes each, and embedded in paraffin wax and cut in 5 µm thin slices using a rotary microtome (Humason 1972). The sections were stained with Eosin and mounted in DPX. Sections were examined using a light and phase contrast microscope (Olympus CX-41). Different parts of the salivary gland were micro-photographed with an E-420 Olympus digital camera with appropriate magnifications.

**Table 1.**
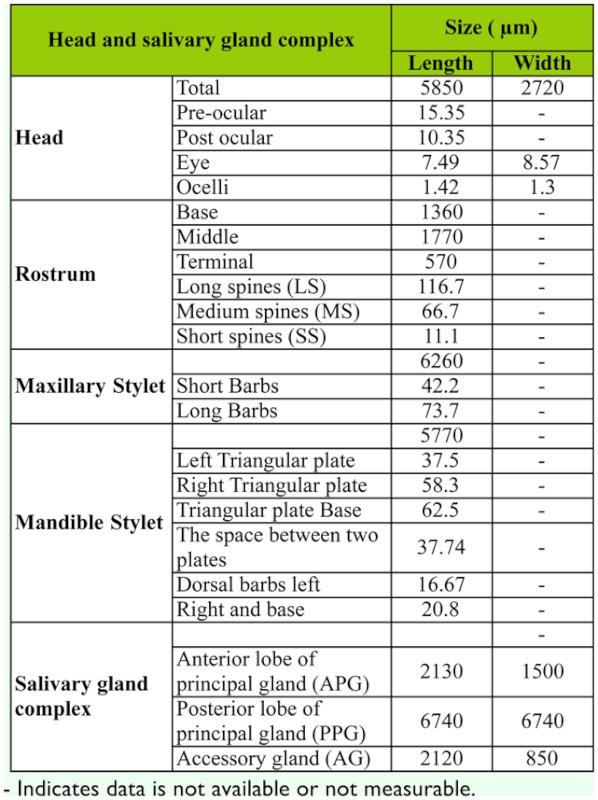
The morphometry measurements (µm) of head, rostrum, stylets and salivary gland complex of *Rhynocoris marginatus* adults.

**Figure 1.**
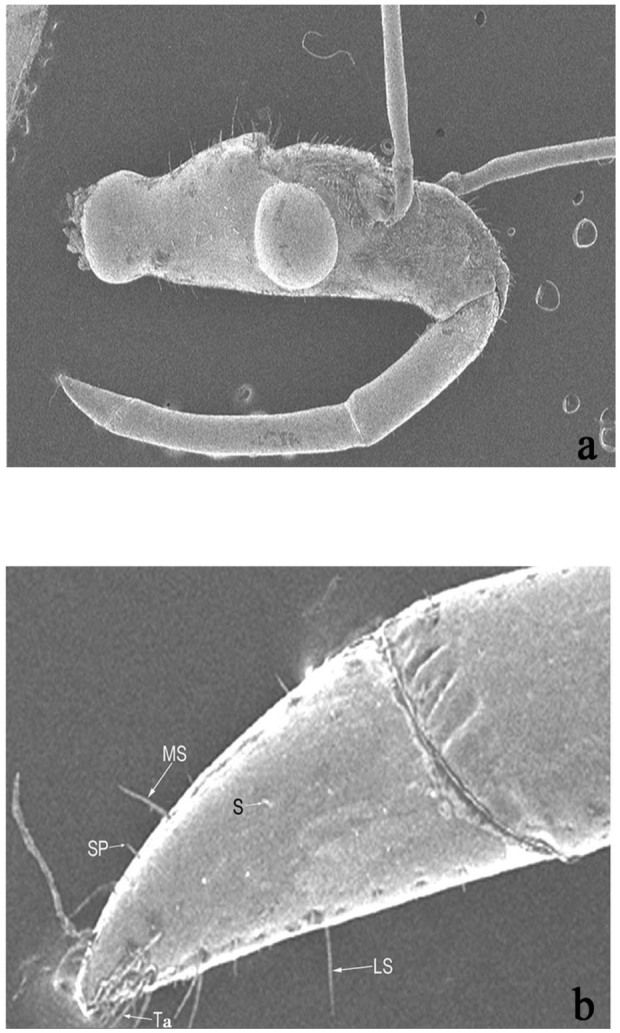
*Rhynocoris marginatus* SEM photographs of head (a) and rostral segment (b). L.S - long spikes, MS median spikes, S - soli, SP short spikes, Ta - trichobothria. High quality figures are available online.

## Results

### Gross morphology of the head, rostrum, and stylet

**Head.** The head is more or less elongate, mobile, and immersed into the eyes. The long and narrow head of *R. marginatus* holds the segmented proboscis, which is used to prey on its victims (Figure 1a). The anteocular area is shorter than the postocular area. Generally, the head is triangular, longer rather than broad, and is often freely movable (Figure 1a). The head component has the following morphometry: post ocular length 10.35 µm; pre-ocular length 15.35 µm; eye length 7.49 µm; eye width 8.57 µm; ocelli length 1.42 µm; ocelli width 1.30 µm.

**Figure 2.**
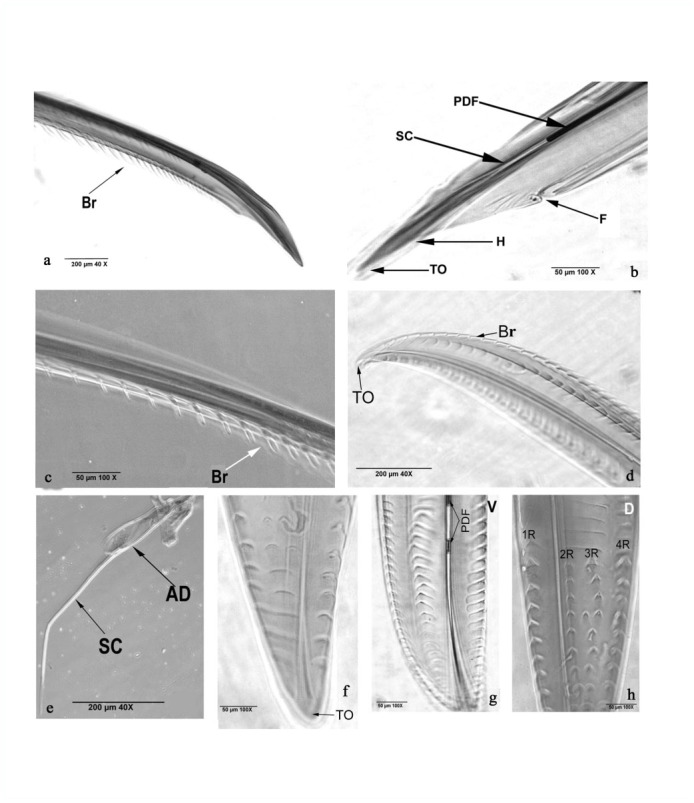
*Rhynocoris marginatus* phase contrast photographs of lateral (a and b) and terminal (c) view of maxillary stylet, magnified tip region of mandibular stylet (d), terminal region with adductor muscle (e), ventral (f and g) dorsal (h) view of mandibular tip. AM adductor muscles, Br - brush-like, F - furrow, H - hook PDF partially digested food material, 1R - first row, 2R - second row, 3R - third row and 4R - fourth row. SC - salivary canal, TO - terminal opening. High quality figures are available online.

**Rostrum.** *Rhynocoris marginatus* has a three—segmented curved rostrum; the middle segment is longer than the base and the terminal segment (Table 1) (Figure 1a). The terminal rostral segment bears three types of fine sensilla: long spines (LS), medium spines (MS), and short spines (SS) (Figure 1b, Table 1). In addition, the tip of terminal segment has trichobothria (T).

**Stylet.** The stylet is formed as a bundle of four long hair-like structures having sharp end at the tip; the base is attached to the head with the help of adductor muscles (AM). The stylet bundle of *R. marginatus* has each a pair of maxillary (6260 µm) inside and mandible stylets (5770 µm) outside. The mandibular stylet, like the maxillary stylet, is attached with adductor muscles (AM). The maxillary stylet is spear-shaped and its tip is highly pointed (Figure 2a) followed by a narrow tube. The junction between the tube and the tip has a furrow (F) that is present on both sides. It is connected with the central salivary canal (SC) (Figure 2b). The right side has 28 strongly curved brush-like barbs (Br) (Figure 2c, d) pointing away from the head on the lateral sides and 23 barbs on the middle. The sharp brush-like barbs (Br) are present on the lateral margin of the stylet. Barbs are short (42.2 µm) at the tip region and long (73.7 µm) at distal region (Figure 2c, d), with distinct barbs laterally and centrally (Figure 2d). The mandibular stylet has barbs as triangular plates with dimension of left = 37.5 µm, right = 58.4 µm, and base (62.5 µm); it is highly pointed and sharp, and opens distinctly with an opening (TO) (Figure 2d) followed by salivary canal (SC) (Figure 2e) that joins with the digestive canal of the salivary apparatus. Reduviids ingest partially digested food (PDF) through the terminal opening (Figure 2b, f, g). The distance between two plates was 37.4 ± 0.3 µm (Figure 2h). Dorsal view of tip shows four rows of short barbs. These short barbs have dimensions of left = 16.6 ± 0.1 µm, right and base = 20.8 µm. Barbs are arranged in 29.12 ± 0.10 µm distances. The distances between second, third, and fourth row was 24.9 ± 0.1 µm, whereas a 62.4 ± 0.1 µm distances was measured between the first and second row. All the barbs in these four rows (1R, 2R, 3R, 4R) are pointed towards the head (Figure 2h). The SC is extended up to the main salivary duct. However, the distal end of the maxillary stylet is attached into the head by adductor muscles (AM) (Figure 2e). Maxillary and mandibular stylets are equally useful for the feeding process.

**Figure 3.**
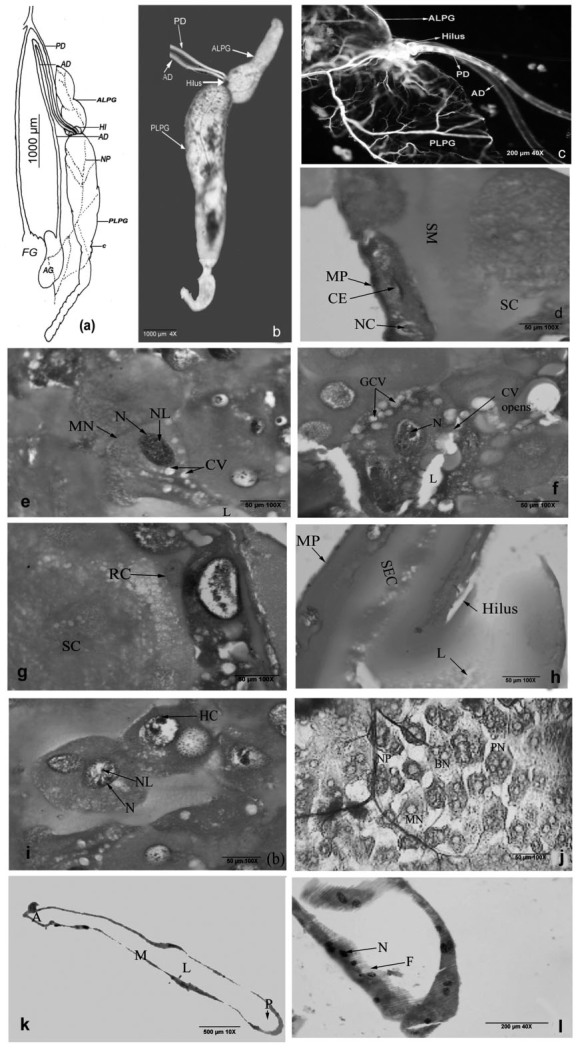
Gross morphology of *Rhynocoris marginatus* salivary gland - line diagram (a) principle gland (4×) (b), hilus region (c), anterior part of the posterior lobe (d), secreting cells of posterior lobe (e), collecting vacuoles of the posterior lobe (f), replacement cells and secretions (g), hilus cross section (h), posterior lobe double nucleated cells (i) principal gland surface cells (j), longitudinal section of accessory gland (k), lower portion of accessory gland (I). (A anterior region, ALPG -anterior lobe of principle gland, AD accessory duct, AG - accessory gland, BN - binucleated, CE - cubic epithelium, CV opens- collecting vacuoles opens, GCV - group of collecting vacuoles, HI- hilus, F-filament, HC- heterochromatin, LLumen, M- mid region, MN- mononucleated, MP- membrane probia, N - nucleus, NL - nucleolus, NP - nerve plexus, P- posterior region, PLPG - posterior lobe of principle gland, PN - polynucleated, SC secretory epithelium, Sc - secretions in dense, SM - secretory materials TN - tirnucleated, T - trachea, To - tracheoles). High quality figures are available online.

### Gross morphology of salivary gland

The salivary gland complex of *R. marginatus* consists of a pair of principal glands (PG) with two lobes and a pair of accessory glands. The principal glands are present on either side of the oesophagus and extend to the anterior crop region. The principal gland is simply bilobed, comprised of the short, triangular anterior lobe of principal gland (APG), and the posterior lobe of principal gland (PPG) (Figure 3a, b) (Table 1). The posterior lobe is highly nodulous at the posterior side, rather than the anterior side of posterior principal gland. These nodules are distinct as constrictions (C). Both principal and accessory glands and their ducts receive a tracheal (T) supply (Figure 3c). This is derived from a tracheal trunk of the first spiraclular trachea. A distinct nerve plexus (NP) is found on the principal gland. This nerve is derived from the hypocerebral ganglion of the stomatogastric nervous system.

The junction of the anterior and posterior lobe has a well developed, compartmentalized hilus (HI) (Figure 3c), provided with valvular openings for the regulation of secretions sent out from different lobes of the main and accessory gland. Deeper constriction is present in the hilus region, and the principal duct (PD) and accessory duct (AD) emerge from the gland in this region (Figure 3c). These two ducts leave the gland at its anterior extremity where PD joins the salivary ducts and leads into stylets, whereas AD runs backwards to join the accessory gland (AG). The accessory gland is vesicular in nature and ovoid in shape. The accessory gland duct (AD) passes forward from the accessory gland into the posterior region of the head where it then turns back and returns to the thorax to join the principal duct as it emerges from the hilus of the principal gland.

### Histology of salivary gland

The bilobed principal gland of *R. marginatus* comprises a single layer of cuboid epithelial cells (CE) with spherical nuclei (NC). Cuboid epithelial cells (length 74.9 ± 2.1 µm; width 16.6 ± 0.5 µm) (Figure 3d) surround membrane probia (MP) that encloses a spacious cavity, where the secretion is stored before being discharged. The cytoplasm is highly viscous with numerous secretory granules (SG) and vacuoles. Both the anterior and posterior lobe cells differ slightly in texture. In the former, vacuoles are moderate and numerous, whereas in the latter the vacuoles are very small and scattered in nature. Mononucleated (MN) (length 24.9 ± 0.5 µm; width 16.6 ± 0.5 µm; nucleus 8.3 ± 0.1 µm diameter), binucleated (BN) (length 24.9 ± 0.5 µm; width 24.9 ± 0.5 µm; nucleus 6.2 ± 0.1 µm diameter), trinucleated (TN) (length 24.9 ± 0.5 µm; width 24.9 ± 0.5 µm; nucleus 4.1 ± 0.1 µm diameter) and polynucleated (PN) (length 33.3 ± 0.6 µm; width 33.3 ± 0.3 µm; nucleus 8.3 ± 0.1 µm diameter) cells are distributed both in the anterior and posterior lobes of the principal gland. Cells present in the anterior principal lobes have less viscous granular cytoplasm than anterior lobes. These CV decrease in size towards the posterior principal gland (Figure 3e). These cells are found in proximity to another binucleated cell or mononucleated cell. The lumen (Lu) of the posterior lobe is narrow (Figure 3f), whereas in the anterior principal gland it is broader. Irregularly shaped secretion granules (SG) are distributed both in anterior and posterior principal glands, which are concentrated in the APG and more separately distributed in the PPG. The posterior lobe has collecting vacuoles (CV), dens secretion (SC), and a group of collecting vacuoles (GCV), as well as replacement cells (RC) and secretions (Figure 3f and g).

Histological features of the hilus (Figure 3h) and posterior regions are presented in Figure 1i and 1j. Mono- (MN), bi- (BN), tri- (TN), and polynucleated cells (PL) are more prominent. As seen in the anterior lobe, the posterior lobe is also surrounded with membrane probia (MP), followed by secretary epithelium (SE) and lumen (LU) (Figure 3h). The different cell types have prominent nucleus (N), nucleus (NL), and heterochromatin (HC) (Figure 1i). The two—lobed heterochromatin (HC) (8.32 ± 0.01 µm diameter) are binucleated with denser secretary materials at anterior principal gland (Figure 3i). The cytoplasm is traversed by large collecting vacuoles (CV) (6.24 ± 0.02 µm diameter) containing secretions.

Histologically, the accessory glands (Figure 3k and 3l) differ distinctly from the principal glands and always produce watery saliva. Accessory glands are formed of a single layer of glandular cells surrounded externally by a basement membrane. Accessory glands are divided into anterior (A) and posterior regions (B). The glandular cells are flat with a small round nucleus devoid of any secretion granules and inclusions. The inner margins of the cells exhibit distinctly striated or separate filaments (F) with a prominent nucleus (N). The vesicular part of the accessory salivary gland is distinctly glandular and possesses a moniliform tubular secretory appendix and lies closely to the posterior region of the crop and the posterior midgut.

## Discussion

The gross morphological features of the mouthparts of *R. marginatus* are similar to other reduviid predators (Sahayaraj et al. 2010). The three-segmented rostrum of *R. marginatus,* like other Harpactorinae, is long and slender and capable of considerable forward extension (Ambrose 1999), which helps to inject the venomous saliva on the nerve fibers directly to paralyse the victims quickly.

The three-segmented rostrum bears a moderate number of mechano and chemosensory hair-like sensillae. It is used in the perpendicular orientation of the stylet fascicle to the prey, which is a typical behavior of many other predatory hemipteran species (Sahayaraj et al. 2010). The hair-like sensillae are used to detect the suitability/palatability/acceptability of the prey. Like all other heteropterans, the stylet bundle of *R. marginatus* has two maxillary stylets inside and two mandibular stylets outside. Heteropteran stylets form a fascicle composed of two lateral mandibular stylets and two maxillary stylets, the former is armed variously with teeth or rasps, and the latter interlocks and forms the salivary and food canals (Cobben 1978); these characteristics have also been observed in *R. marginatus.* Boyd Jr. et al. (2002) and Boyd Jr. (2003) reported that the maxillary stylets of predatory mirids are more serrated than the phytophagous insects, but the mandibular stylet has deeper serrations in *R. marginatus,* probably used to disrupt prey by ripping and tearing host tissues (Cohen 2000). The barbs are pointed away from the head, indicating that the cutting action occurs when the stylet is thrust forward. Unlike predacious pentatomids, reduviid predators have barbs on the mandibular stylets pointing toward the head as reported by Cohen (2000). The presence of mandibular and maxillary stylets (barbs) help to deeply penetrate the tissue and pump toxic saliva into the prey, under turgor pressure. The broader side of triangular barbs in mandibular stylets helps by allowing the partially digested food from going out into the salivary canal. The bundle is housed in the labium and stabilized by the labium. The interlocking mechanism of the mandibular and maxillary stylets inside the rostrum forms food canals continuous with the salivary canal. Presence of partially digested food (Figure 3b, 3g) indicates that both mandibular and maxillary stylets sucked out liquefied food from the host.

The anatomical pattern of the salivary system of *R. marginatus* conforms to the general heteropteran plan (Southwood 1955), and reduviids in particular (Louis and Kumar 1973; Haridass and Ananthakrishnan 1981; Morrison 1989; Azevedo et al. 2007). The salivary system has a pair of PG and another tubular accessory gland as found in reduviids (Haridass and Ananthakrishanan, 1981; Maran, 1999; Sahayaraj et al. 2010). The principal gland of *R. marginatus* has an anterior and a posterior lobe as observed in Peiratinae (Sahayaraj et al. 2010), Reduviinae, Salyavatinae, and Harpactorinae (Haridass and Ananthakrishnan 1981) of Reduviidae. Louis and Kumar (1973) suggested the trilobed condition of the salivary system as a primitive type, reduced to the bilobed condition as observed in reduviids; an advanced character and unilobed condition was most advanced in Triatominae of Reduviidae (Anhê and Oliveria 2008). The principal salivary glands of *R. marginatus* are elongated vesicles with tubular extensions as observed among the members of Reduviinae, Salyavatinae, and a member of Harpactorinae (*Sycanus collaris*) (Haridass and Ananthakrishnan 1981). The differential functions of anterior and posterior lobes suggest division of labor, though Baptist (1941) believed that there was no such division in the functions of salivary glands of Heteroptera. In Pentatomomorphid families, the secretions of anterior lobes are primarily concerned with stylet-sheath formation, whereas those of posterior lobes are involved in the production of digestive enzymes (Hori 1969; Miles 1972). Edwards (1961) found the presence of zootoxic enzymes in both the anterior and posterior lobes of *Platymeris rhadhamanthus.* The secretion in the anterior lobe is lesser in quantity, viscous, and transparent, whereas the posterior lobe secretes a larger quantity of highly viscous and milky white secretions as reported by Haridass and Ananthakrishnan (1981).

A membrane propia is very well marked in the PG. The muscle layer associated with the lobes was not found in the PG, which indicated that the saliva of *R. marginatus* could be injected into the prey by extrinsic muscles. The presence of muscle fibers seems to be related with the predatory habit (Baptist 1941), where a muscle sheath might be important to mobilize greater amounts of saliva. The nerve plexus, trachea, and fine muscle fibers support and discharge the saliva needed for paralyzing or killing the prey. The tracheal supply of the salivary gland comes from the first visceral trachea as observed by Baptist (1941). The AG of *R. marginatus* is typically of the vesicular type (Edwards 1961), especially in other harpactorines, reduviines, and salyavatines (Haridass and Ananthakrishnan 1981; Vellingirinathan 1986; Agnes 1990). Accessory glands are filled with watery fluid, which helps the predator to flush out the predigested food from the body of the prey, very similar to the lacerate-flush mode of feeding in Pentatomomorpha (Miles 1972; Miles and Slowiak 1976).

The hilus is distinct in *A. pedestris* (Morrison 1989). It provides a regulatory system for delivery of secretions from different lobes of the salivary system. In *Lestomerus affinis* and *Haematorrhophus nigroviolaceous*, the valves in the hilus make it possible not only to send the secretions independently from the AG, but also to send the secretions separately from the anterior and the posterior lobes of the principal gland (Haridass and Ananthakrishnan 1981). Such an independent flow for the anterior and the posterior lobes of the principal gland is also observed in *R. marginatus* (Figure 3c).

In *R. marginatus,* we recorded mono-, di-, tri-, and polynucleated cells both in the anterior and the posterior lobe of the principal gland. Usually, each glandular cell possesses one or more distinct nuclei. The nucleus is large, mostly rounded in shape, and occupies a major portion of the cytoplasm. It possesses one or two large and irregular nucleoli, while the chromatin is in the form of minute microsomes distributed more or less uniformly in the nucleus. Both the anterior and posterior lobes possess binucleated cells. But Morrison (1989) observed uninucleate cells in the anterior lobe and binucleate cells with highly viscous cytoplasm in posterior lobes of *Acanthapis pedestris.* Such variations are found among members of different subfamilies of Reduviidae.

The cytoplasm is traversed by various sizes of collecting vacuoles (Cv) containing secretions. The size of the collecting vacuoles increases towards peripheral as well as towards the lumen of the PG. Regular, rounded secretion granules are distributed to near or around the collecting vacuoles. Another characteristic feature is that the central lumen of the gland is lined by a special flattened secretory epithelium, with irregular intercellular space around the central lumen. The cytoplasm possesses typical secretion granules and is dense around the collecting vacuole. These characteristic features of collecting vacuoles are no doubt developed as an adaptation to the large size of the cell, and they serve the purpose of storing up quite an appreciable quantity of secretion. It must be assumed that secretion granules are typically built up in cytoplasm to the zymogen-granule stage, and are then stored as such in the cytoplasm. A well-developed nerve plexus is always present on the surface of the PG, though in many cases the nerves that supply this plexus were not traced owing to the minute size of the whole system. Presumably they must be present.

The salivary system is richly supplied by nerves from the sub-esophageal ganglion and stomatogastric system, as reported by Miles and Slowiak (1976). Moreover, a pair of nerves separately connected to the anterior and posterior lobes facilitates independent discharge of saliva (Miles 1972). However, a poorly developed nervous plexus is always present on the surface of the AG. Despite their similarity in morphology (Haridass and Ananthakrishnan 1981), there are some histological variations in the salivary glands of *R. marginatus.*

The AG attached to the lateral sides of the first midgut appears triangular with a tubular appendix opening into the common salivary duct. Accessory glands are thought to function as water recapturing organs, a function that has been underemphasized in an account of feeding by predacious heteropterans (Miles 1972). Accessory salivary glands are filled with watery fluid (Baptist 1941) that recirculates water from the gut to ensure a copious flow of watery saliva, helping the predator to flush out predigested food from the body of its prey. The fluid is forwarded by a single-layered epithelium as observed in other predatory bugs like *Brontocoris tabidus* (Azevedo et al. 2007). The AGs differ histologically from the lobes of the PG and secrete watery saliva, which has less protein fractions than the other lobes. Similar results were highlighted in Pentatomids and Coreids (Miles and Slowiak 1976), and in assassin bugs (Haridass and Anathakrishnan 1981; Morrison 1989).

In conclusion, this study revealed a number of interesting aspects related to morphology and histology of mouthparts of *R. marginatus.* We described the gross morphology and rostrum, mandible, maxillae, and salivary gland complex of *R. marginatus* in detail, along with sixteen salivary complexes reported by Haridass and Ananthakrishnan (1981). The functional morphology of *R. marginatus* salivary gland, head, and mouthparts have been adapted to support the predatorory habits of this predator.
